# Green HPLC-PDA method for simultaneous determination of linagliptin and cefixime with pharmacokinetic application in rats

**DOI:** 10.1038/s41598-026-57925-0

**Published:** 2026-07-07

**Authors:** Weam M. Othman, Nehal F. Farid, Nourah Z. Al-zoman, Ibrahim A. Darwish, Samah S. Saad, Fatma F. Abdallah

**Affiliations:** 1 Pharmaceutical Analytical Chemistry Department, Faculty of Pharmacy, Misr University for Science and Technology, 6th October City, Egypt; 2 Pharmaceutical Analytical Chemistry Department, Faculty of Pharmacy, Beni-Suef University, Beni-Suef, Egypt; 3 Department of Pharmaceutical Chemistry, College of Pharmacy, King Saud University, P.O. Box 2457, 11451 Riyadh, Saudi Arabia

**Keywords:** Type-2 diabetes, Linagliptin, Cefixime, High performance liquid chromatography, Pharmacokinetic studies, Therapeutic drug monitoring, Chemistry, Drug discovery, Medical research

## Abstract

Many individuals with diabetes have compromised immune systems and reduced peripheral sensation, making them susceptible to infections. As a result, combination therapy involving antidiabetic drugs and antibiotics has become essential. A newly introduced combination of the antidiabetic drug linagliptin (LIN) and the third-generation cephalosporin antibiotic cefixime (CEF) has been recommended to combat infections in diabetic patients.To optimize therapeutic efficacy and minimize adverse effects associated with this combination therapy, a reliable analytical method was essential for pharmacokinetic analysis and therapeutic drug monitoring. This study present, for the first time, a green high-performance liquid chromatographic method with photodiode array detection (HPLC-PDA) for the simultaneous determination of LIN and CEF in plasma samples. Chromatographic separation was achieved using a Symmetry C_18_ column (250 mm × 4.6 mm, 5 μm particle size) under isocratic elution with a mobile phase consisting of 20 mM sodium phosphate (pH 4.3, adjusted with orthophosphoric acid) and methanol (50:50, v/v). The flow rate was set at 0.8 mLmin^− 1^, with a total run time of 12 min. The injection volume was 20 µL and the detection was performed at 230 nm. The method demonstrated linearity over a range of 50–2000 ng mL^−1^for both LIN and CEF, with limits of detection (LOD) of 24 and 21 ngmL⁻¹ and limits of quantitation (LOQ) of 43 and 45 ngmL⁻¹ for LIN and CEF, respectively. Validation parameters complied with ICH M10 bioanalytical guidelines. Additionally, the method was successfully applied to a pharmacokinetic study comparing drugs efficacy when administered alone versus concurrently. The results demonstrated that co-administration of LIN and CEF significantly altered their bioavailability: LIN C_max_ increased by 63.2% and AUC increased by 134.2%, while CEF C_max_ decreased by 27.3% and AUC decreased by 23.3%, indicating a bidirectional pharmacokinetic interaction, and underscoring the need for careful monitoring during combination therapy.The greenness of the proposed HPLC-PDA method was evaluated using four metric tools and the findings confirmed the method’s minimal environmental impact. In conclusion, the developed HPLC-PDA method not only provides a reliable tool for therapeutic drug monitoring in clinical practice but also establishes a robust framework for future investigations into drug-drug interactions in human therapeutics.

**Supplementary Information:**

The online version contains supplementary material available at 10.1038/s41598-026-57925-0.

## Introduction

**Fig. 1:**
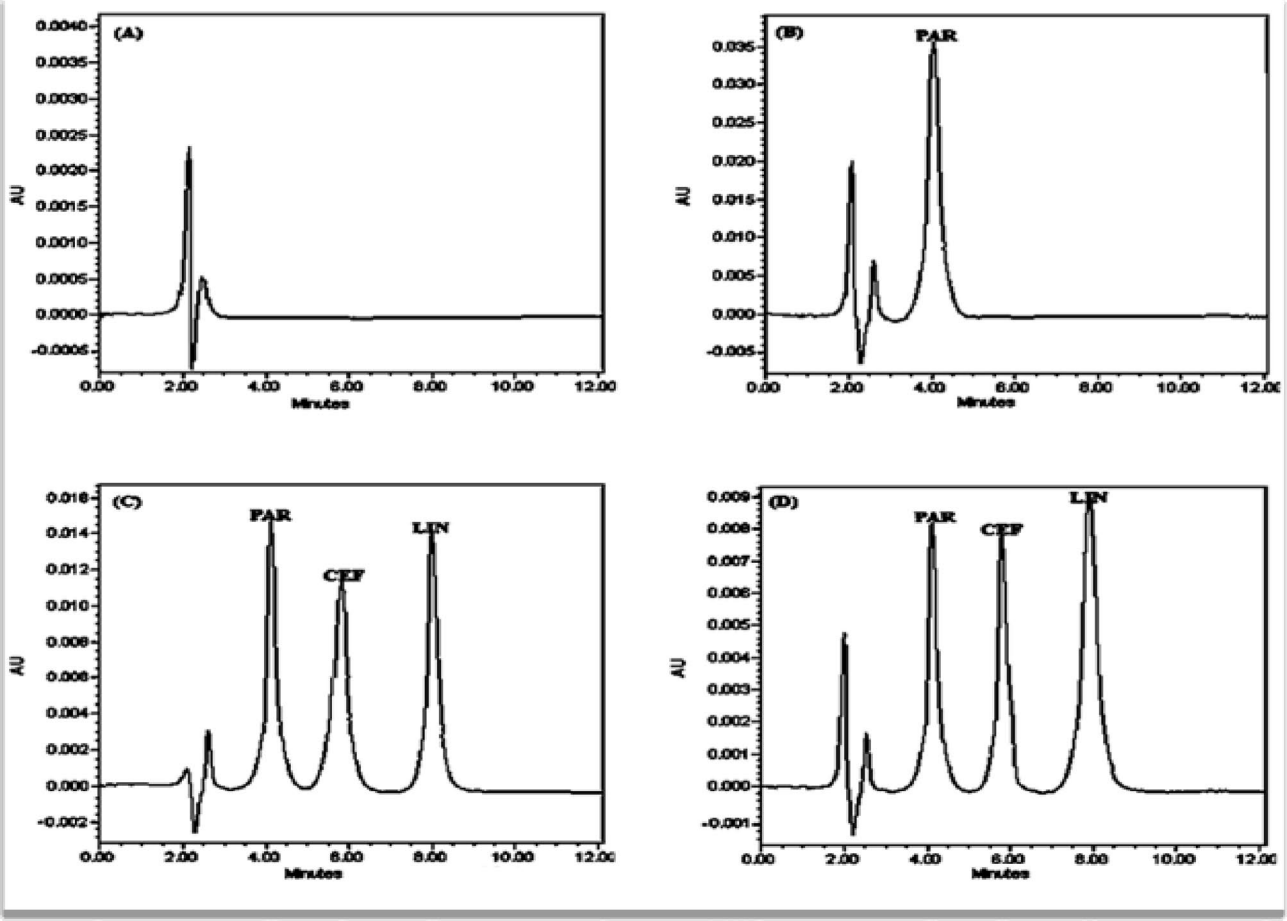
Representative HPLC-PDA chromatograms demonstrating method selectivity. (**A**) Blank rat plasma showing no endogenous peaks at the retention times of the analytes or internal standard. (**B**) Blank rat plasma spiked with 1000 ngmL^-1^ PAR (internal standard) showing retention time at 3.95 min. (**C**) Rat plasma spiked with PAR (1000 ngmL^-1^), cefixime (800 ngmL^-1^, RT = 5.64 min), and linagliptin (900 ngmL^-1^, RT = 7.96 min), demonstrating baseline separation with resolutions of 4.38 (plasma/PAR), 3.76 (PAR/CEF), and 5.16 (CEF/LIN). (**D**) Real rat plasma sample collected 3 h after oral co-administration of LIN (10 mg/kg) and CEF (8 mg/kg), containing LIN (1102 ngmL^-1^) and CEF (722 ngmL^-1^) with PAR (100 µL). Chromatographic conditions: Symmetry C_18_ column (250 × 4.6 mm, 5 μm); mobile phase: 20 mM sodium phosphate buffer (pH 4.3): methanol (50:50, v/v); flow rate: 0.8 mLmin^-1^; detection: 230 nm; temperature: 25 °C.

Diabetes mellitus represents a rapidly growing global health crisis, imposing substantial social, healthcare, and economic challenges^1^. The absence of effective prevention or treatment strategies is expected to lead to a significant increase in diabetes prevalence, thereby rendering antidiabetic medications crucial for patient survival. Linagliptin (LIN, Fig. S1) is an orally administered, highly selective dipeptidyl peptidase-4 inhibitor^2^ utilized in the management of type 2 diabetes mellitus. It improves glycemic control, whether used alone or in conjunction with other antidiabetic medications^2^. Patients with diabetes face increased susceptibility to infections, which can exacerbate disease management challenges^3^. Common complications encompass immune system dysfunction, periodontal disease, cardiovascular disorders, diabetic foot infections, external otitis, rhino cerebral mucormycosis, and emphysematous pyelonephritis^3^. Effective treatment generally necessitates the use of broad-spectrum antibiotics in conjunction with comprehensive clinical evaluation and monitoring^1–3^. Cefixime (CEF, Fig. 1), a third-generation cephalosporin antibiotic, demonstrates broad-spectrum antibacterial activity^4^ and remains one of the most frequently prescribed antibiotics for diabetes-related infections^5,6^. While specific clinical trials evaluating the LIN-CEF combination are not yet available, the co-administration is clinically rationalized based on: (i) the increased susceptibility of diabetic patients to bacterial infections requiring broad-spectrum antibiotic coverage^3^, (ii) the established safety profiles of both agents when used individually^2,4^, and (iii) published reports of cefixime co-administration with other antidiabetic agents such as metformin and glimepiride^7,8^. The present study provides the first analytical validation and pharmacokinetic characterization of this specific combination.

Furthermore, mechanistic plausibility for a pharmacokinetic interaction is supported by the fact that LIN is a known substrate of P-glycoprotein (P-gp) and CYP3A4, while CEF has been reported to interact with P-gp and organic anion transporting polypeptides (OATPs), suggesting potential for transporter-mediated competition when both drugs are co-administered.

LIN has been quantified using various analytical approaches, including RP-HPLC^9–11^, spectrophotometry^12^, spectrofluorimetry^13^, colorimetry^14^, voltammetry^15^, UPLC-MS/MS^16,17^, HPTLC^10,11^, and capillary electrophoresis^18^. Similarly, CEF has been analyzed by multiple techniques, including HPLC-UV^19,20^, LC-MS^21^, spectrophotometry^22^, spectrofluorimetry^23^, colorimetry^24^, voltammetry^25^, RP-HPTLC^26^ and electrophoresis^27^.Although all these methods can quantify LIN or CEF individually, only one method has been reported for their simultaneous in vivo determination^28^. Unfortunately, this method lacked the sensitivity required for comprehensive pharmacokinetic analysis. To address this limitation, a more sensitive analytical approach was needed to fully characterize the pharmacokinetics of both drugs—when administered in combination. This need is also critical for therapeutic drug monitoring (TDM) in patients receiving combined LIN-CEF therapy. Effective TDM enables personalized dosing to optimize treatment outcomes—balancing infection control and glycemic management while minimizing risks such as LIN-induced hypoglycemia. Additionally, precise monitoring of CEF exposure helps prevent antibiotic resistance. Given these clinical imperatives, the development of a robust analytical method was essential to bridge existing gaps in pharmacokinetic analysis and TDM for LIN-CEF combination therapy.

The HPLC-PDA has been demonstrating unique practical advantages as it compromised as balance between analytical performance and practical utility. Unlike specialized techniques like LC-MS/MS that require expensive instrumentation, HPLC-PDA offers clinically relevant sensitivity at a fraction of the cost, making it accessible for routine pharmacokinetic studies and TDM. Its PDA detector provides superior selectivity for simultaneous drug quantification without interference from biological matrices^29^. These advantages position HPLC-PDA as an ideal platform to address the urgent need for monitoring LIN-CEF interactions while maintaining compliance with ICH M10 guidelines^30^. Importantly, the achieved LOQ values (43 ng mL⁻¹ for LIN and 45 ng mL⁻¹ for CEF) are well below the expected C max values following therapeutic oral doses (950–1550 ng mL⁻¹), confirming that the method possesses adequate sensitivity for the intended pharmacokinetic application without recourse to more expensive LC-MS/MS instrumentation.

This study describes the first HPLC-PDA method for the simultaneous quantification of LIN and CEF in plasma samples at nanogram levels. The method was optimized and rigorously validated in compliance with ICH M10 bioanalytical guidelines^30^, demonstrating successful application in analyzing real plasma samples from rats administered both LIN and CEF. This innovative approach offers multiple analytical advantages: (1) a simple one-step protein precipitation for plasma sample preparation, (2) minimal sample volume requirements, (3) reduced solvent consumption, and (4) rapid analysis (run time of < 12 min). These features collectively enable high-throughput processing, making the method particularly suitable for clinical laboratory implementation. Notably, the method aligns with Green Analytical Chemistry (GAC) principles through its eco-friendly design. As the first reported HPLC-PDA method for simultaneous LIN-CEF quantification, it establishes a valuable platform for pharmacokinetic studies, bioavailability assessments, and TDM in diabetic patients receiving this combination therapy.

## Experimental

### Apparatus

Waters 2695 Alliance HPLC system equipped with G 1311 C quaternary pump, G 1322 A on-line degasser, G 1329B auto-sampler, G 1316 A thermo stated column compartment, and 996 photodiode array detector. A pH meter, model NV P-910 (Consort, Belgium) was utilized for adjusting pH values of buffer solutions used throughout the study. A digital balance, model JB1603-C/FACT, was a product of Mettler-Toledo International Inc. (Zürich, Switzerland). Pure water was obtained through Purelab Flex water purification system (ELGA Veolia Ltd., High Wycombe, UK) throughout the study.

### Materials, reagents, and experimental animals

The reference standard materials of LIN and CEF were purchased from LC Laboratories (Woburn Massachusetts, USA) with purity of > 99%. Paracetamol (PAR) was obtained from Jigs Chemical Limited (Ellisbridge, Ahmedabad, India) with purity of > 99%.HPLC grade solvents (methanoland acetonitrile) were purchased from Fischer Scientific (London, United Kingdom). Analytical grade sodium dihydrogen phosphate, orthophosphoric acid and sodium hydroxide were purchased from Merck (Darmstadt, Germany). Phosphate buffer solution (20mM, pH 4.3) was prepared by dissolving 0.18 g of sodium phosphate monobasic in approximately 90 mL deionized water, completing the volume to 100 mLwith deionized water, and adjusting the pH to 4.3 using 0.1 N orthophosphoric acid. Membrane filters (0.2 μm) were purchased from Nihon Millipore (Yonezawa, Yamagata, Japan). All other materials used throughout this study were of analytical grade.

Twenty eight healthy male Wistar Albino rats, weighing 250 ± 30 g were obtained from the animal facility at the College of Pharmacy, King Saud University (Riyadh, Saudi Arabia) where all subsequent animal housing, dosing, and sample collection procedures were conducted. The animals were observed on a daily basis to ensure their ongoing wellness. All rats were housed in standard laboratory cages under controlled environmental conditions:12-hour light/dark cycle, ambient temperature maintained at 24–27 °C, relative humidity of 40–60%, and adequate ventilation.

Following a 7-day acclimatization period to the laboratory environment, animals were fasted overnight (12 h) with free access to water prior to experimental procedures. The 7-day period was selected based on established laboratory animal acclimatization standards (Suckow et al., The Laboratory Rat, 2nd Edition, Elsevier, 2012), which specify a minimum of 5–7 days for physiological stabilization following transport. Health monitoring during this period included daily assessment of body weight (variation ≤ 5% over 7 days), general appearance (fur smooth, eyes clear, no discharges), behavioral activity (normal grooming, feeding, ambulation), respiratory status (no wheezing or labored breathing), and stool consistency (formed, normal color). All animals met these criteria prior to study initiation.

In our pharmacokinetic study, the animals were maintained in a state of consciousness for the duration of the experimental period to accurately reflect the systemic drug exposure profile following oral administration and to ensure accurate pharmacokinetic assessment, as anaesthesia can alter drug absorption, metabolism, and physiological parameters^31^.

For the purpose of collecting blood samples at various time points, the rats were briefly sedated using a non-invasive inhalation method. Specifically, the animals were placed in a closed chamber and exposed to isoflurane gas for a minimal duration, rendering them sufficiently calm to allow for safe handling and collection of blood samples via the tail vein. The animals were returned to their cages immediately after sampling and regained full consciousness within seconds, thus minimizing any potential stress or impact on the study’s pharmacokinetic end points. This approach was chosen to ensure the welfare of the animals while maintaining the integrity of the data.

The study was approved by the Institutional Animal Care and Use Committee (IACUC), Faculty of Pharmacy, Beni-Suef University, Egypt (Approval No. REC-H-PhBSU-022-234). At the conclusion of the study, animals were humanely euthanized in accordance with approved institutional animal care protocols. Euthanasia was performed using a two-step method to ensure no pain or distress. First, the animals were deeply anaesthetized with a lethal dose of a ketamine (80 mg/kg) and xylazine (10 mg/kg) mixture administered via intraperitoneal injection to induce deep anesthesia. This was immediately followed by a secondary physical method of euthanasia, such as cervical dislocation, while the animals were completely unconscious. This dual methodology is consistent with and adheres to the ethical standards outlined in the AVMA Guidelines for the Euthanasia of Animals^32^. All procedures were conducted in compliance with institutional and international ethical standards, including the ARRIVE guidelines^33^.

### Preparation of standard solutions

LIN, CEF, and PAR standard stock solutions were individually prepared by dissolving 10 mg of each compound in separate 10-mL volumetric flasks, with methanol added to reach the final volume (1 mgmL^-1^ concentration). Working solutions (100 µgmL^-1^) were then prepared by diluting 1 mL aliquots of each stock solution to 10 mL with mobile phase. All methanolic stock solutions demonstrated stability for at least three weeks when stored at 4 °C.

### Preparation of plasma samples for analysis

For plasma sample preparation, 200 µL of rat plasma was aliquoted into a clean test tube. Protein precipitation was achieved by adding methanol to a final volume of 1 mL, followed by vortex mixing for 30 s. The mixture was then centrifuged at 5,000 rpm (2,500 × g) for 10 min at 4 °C. The resulting supernatant was carefully collected and filtered through a 0.2 μm membrane filter prior to injection into the HPLC sytem for analysis.

### HPLC analysis and construction of calibration curves

The chromatographic separation was achieved on a Symmetry C_18_ column (250 × 4.6 mm, 5 μm) maintained at 25 °C. Amobile phase consisting of 20 mM sodium phosphate buffer (pH 4.3, adjusted with orthophosphoric acid) and methanol (50:50, v/v) was isocratically delivered at 0.8 mLmin^-1^, for a total run time of 12 min. Samples (20 µL injection volume) were injected and the eluted compounds were detected at 230 nm using a PDA detector. Prior to analysis, all samples were homogenized using an IVM-300p vortex mixer (Gemmy Industrial Corp., Taipei, Taiwan). Calibration standards were prepared by transferring aliquots (5-200 µL) of LIN and CEF working solutions (100 µgmL^-1^) into separate test tubes to generate concentration ranges of 50-2000 ngmL^-1^ for both analytes. To each tube, 100 µL of PAR internal standard solution (from the working solution 10 µgmL^-1^) was added, followed by methanol to achieve a final volume of 1 mL (PAR concentration: 1000 ngmL^-1^). All samples were processed in triplicate (20 µL injections) under the optimized chromatographic conditions. Calibration curves were constructed by plotting the peak area ratios (analyte/IS) against the corresponding nominal concentrations (ngmL^-1^). For each drug, quality control samples were prepared at four concentration levels according to ICH M10 requirements: LLOQ QC (50 ngmL^-1^), Low QC (150 ngmL^-1^), Medium QC (700 ngmL^-1^), and High QC (1500 ngmL^-1^). All samples and quality control solutions were stored at -20 °C in the refrigerator until analysis time.

### In-vivo studies in rats

The rats were randomly allocated into four experimental groups (*n* = 7 rats per group). Group I received 10 mg/kg LIN in saline^34^, Group II received 8 mg/kg CEF in 5% DMSO-saline^35^, Group III received combined LIN (10 mg/kg) and CEF (8 mg/kg), and Group IV received Vehicle control solution (saline). All treatments were administered as a single oral gavage dose. Blood samples (∼400 µL) were collected via tail vein into heparinized tubes at predetermined intervals (0.5, 0.75, 1, 2, 3, 4, 5, 6, 8, and 12 h post-administration). The total blood volume collected (~ 4 mL over 12 h) represents approximately 20–25% of the estimated total blood volume (15–20 mL) in a 250 g rat. This is within the acceptable limit for survival studies, as published guidelines recommend not exceeding 15–20% of total blood volume over 24 h when animals have free access to water^36,37^(Diehl et al., 2001; Lee et al., 2020). Each single collection of ~ 400 µL represents 2-2.5% of total blood volume, well below the 10–15% limit for a single collection. Animals were provided with free access to water throughout the study to maintain hydration, and no adverse effects (e.g., lethargy, weight loss, or mortality) were observed. Following centrifugation (5,000 rpm, 20 min), plasma aliquots were stored at -20 °C until analysis. For analysis, plasma samples (200 µL) were spiked with 100 µL of PAR as an internal standard (IS) from a working solution (10 µgmL^− 1^) to reach a final concentration of 1000 ngmL^− 1^ and protein-precipitated with methanol (1 mL as the final volume). After vortex mixing (1 min) and centrifugation (5,000 rpm, 20 min), the clear supernatant was filtered through 0.45 μm syringe filters. All samples were analyzed in triplicate using the validated HPLC-PDA method. Plasma concentration-time profiles were generated, and pharmacokinetic parameters were calculated using PK Solver (a freely available menu-driven tool) and the non-compartmental pharmacokinetic properties of elimination rate constant (k), half-life (t_1/2_), clearance (Cl), total AUC and apparent distribution volume (V_d_) in were calculated by Microsoft Excel software.

## Results and discussion

### Method development and optimization

The photodiode array detector was selected over conventional single-wavelength UV detection for three distinct advantages particularly valuable for bioanalytical applications. First, the PDA enables post-acquisition multi-wavelength analysis, allowing retrospective examination of chromatographic data at any wavelength without sample re-injection which is a critical capability when analyzing complex plasma matrices where endogenous interferences may not be apparent at a single wavelength. Second, the PDA provides peak purity assessment through spectral comparison across the elution profile, enabling detection of co-eluting endogenous matrix components that could compromise quantification accuracy, a risk that cannot be identified with single-wavelength detection. Third, the PDA facilitates unequivocal peak identification via UV spectral matching against reference standards; given that LIN and CEF possess distinct UV absorption characteristics, spectral confirmation adds an orthogonal dimension of selectivity beyond retention time alone.

These capabilities make HPLC-PDA a robust analytical platform for the simultaneous determination of multi-component drug formulations in complex biological matrices, ensuring high specificity even in the presence of matrix interferences. Furthermore, the developed method emphasizes sustainability by optimizing solvent consumption and shortening run times, aligning with green analytical chemistry principles. By combining high sensitivity, selectivity, and eco-friendly attributes, this HPLC-PDA approach is well-suited for pharmacokinetic investigations in rats, offering a reliable tool for therapeutic drug monitoring and biomedical research.

Different experimental conditions were studied, including column type, mobile phase composition, flow rate, detection wavelength, and selection of internal standard to achieve optimum resolution and peak intensity (Table S1).

HPLC paired with a PDA detector is a robust analytical platform for the simultaneous determination of multi-component drug formulations, particularly in complex biological matrices. This technique was selected for the concurrent quantification of LIN and CEF due to its ability to provide both retention time and spectral confirmation, ensuring high specificity even in the presence of matrix interferences. The PDA detector enhances method reliability by enabling peak purity assessment and spectral matching, critical for accurately resolving co-administered drugs with overlapping chromatographic profiles. Furthermore, the developed method emphasizes sustainability by optimizing solvent consumption and shortening run times, aligning with green analytical chemistry principles. By combining high sensitivity, selectivity, and eco-friendly attributes, this HPLC-PDA approach is well-suited for pharmacokinetic investigations in rats, offering a reliable tool for therapeutic drug monitoring and biomedical research.

Different experimental conditions were studied, including column type, mobile phase composition, flow rate, detection wavelength, and selection of internal standard to improve and get the optimum resolution and peak intensity of the resulting peaks (Table S1).

#### Detection wavelength

PDA detector was adjusted at 230 nm based on the reported UV spectra of the three analytes^38–40^, where they were all overlapped. Upon choosing 230 nm as the detection wavelength, the three components can be measured with the highest sensitivity (Fig. S2).

#### Column type

The following stationary phases were systematically evaluated: Thermo Fisher BDS Hypersil C_18_ (100 mm length × 3 mm i.d., 3 μm particle diameter), Nucleosil CN (250 mm length × 4.6 mm i.d., 5 μm particle diameter), Prontosil Kroma plus C_18_ (150 mm length × 4.6 mm i.d., 5 μm particle diameter), Discovery C_8_ (250 mm length × 4.6 mm i.d., 5 μm particle diameter), and Symmetry C_18_ (250 mm length × 4.6 mm i.d., 5 μm particle diameter). The C_8_ column failed to achieve baseline separation between LIN and CEF under the investigated green chromatographic conditions, likely due to reduced hydrophobic retention. Among the C_18_ columns evaluated, the Symmetry C_18_ provided superior performance based on three criteria: baseline resolution greater than 2.0 between LIN and CEF, symmetric peak shapes with asymmetry factors between 0.95 and 1.05, and reproducible retention times across multiple injections. The longer column length of 250 mm provided adequate theoretical plates, while the 5 μm particle size balanced separation efficiency against acceptable back-pressure below 2,000 psi.

#### Mobile phase composition and flow rate

Towards a clean and green environment, preliminary trials were directed towards utilization of ethanol as one of the most available and greenest organic solvents^41–43^. Initial trials were done in an attempt to use green solvents; therefore, ethanol and water were used in different ratios; however, the peaks were broad and unresolved. Ethanol was then changed with methanol; nevertheless, results were the same. Furthermore acetonitrile-water was used in different ratios, but the peaks of both drugs were overlapped.

In an attempt to reduce the run time and improve the shape of the peaks, different pH values were tried by the addition of acid or alkali to the water portion such as 0.1 N NaOH, and glacial acetic acid; however, all these trials were failed neither to improve the peaks resolution nor symmetry. Ion pair reagents as triethylamine and octane sulfonic acid sodium salt were also tried; however, no improvement in the separation has been noticed.Additionally, different buffers with various pH values were tried ranging from (3 to 6) in different ratios either with acetonitrile or methanol, and it was observed that the latter showed better results and sharper peaks as well as being greener than acetonitrile. Different concentrations for phosphate solution was tried as 10, 20, 30 mM; where 20 mM gave sharper peaks and 30 mM gave almost the same result, therefore 20 mM was chosen Additionally Finally, a mixture of 20 mM sodium phosphate solution, pH 4.3 adjusted using orthophosphoric phosphoric acid: methanol, (50:50, v/v) was the optimum tried one, and resulting in sharp symmetrical peaks with a reasonable Rt for all the studied components, as shown in Figs. S2, 1. Furthermore, the selected mobile phase possesses low cut off and low buffer concentration was also utilized to minimize column back pressure and salting out.

Flow rates of 0.4, 0.8, 1.0, and 1.2 mLmin^-1^ were evaluated. At 0.4 mLmin^-1^, the run time exceeded 20 min with significant peak broadening (peak width at half-height > 0.3 min), which would compromise throughput and sensitivity. At 1.0 and 1.2 mL/min, peak asymmetry increased markedly with asymmetry factors exceeding 1.2, and resolution between LIN and CEF deteriorated below 1.8, likely due to insufficient mass transfer kinetics at elevated flow rates. The flow rate of 0.8 mLmin^-1^ produced optimal results with sharp, symmetric peaks (asymmetry factor ≤ 1.05), baseline resolution (Rs > 2.0), and a practical run time of 12 min. This flow rate also minimized column back-pressure to approximately 1,600 psi, preserving column longevity. Consequently, the flow rate of 0.8 mLmin^-1^ was selected as optimum (Figs. S2, 1).

#### Selection of internal standard

Different internal standards were tried; including sitagliptin, naproxen, naphazoline hydrochloride, diclofenac potassium, ciprofloxacin, paracetamol and ibuprofen. The most suitable internal standard was paracetamol (PAR) in terms of resolution from the studied drugs and peak plasma.

#### System suitability parameters

All the system suitability parameters were studied and calculated accurately as shown in Fig. S2 and all the obtained results lied within the reference values of the United State Pharmacopeia^44^ as shown in Table S2.

### Method validation

The HPLC-PDA method underwent validation in adherence to the ICH M10 (2022) guideline for bioanalytical method validation^30^, in terms of the parameters outlined below.

#### Linear range and sensitivity

To assess the linearity of HPLC-PDA method, a set of 10 concentration levels of samples were analyzed and used to generate the calibration plots. Linear regression analysis of the data set was conducted; linear fitting equations and their correlation coefficients were computed. The linearity of HPLC-PDA for LIN and CEF was evaluated using the correlation coefficients, as measures, within the specified concentration ranges. A summary of the calibration parameters for both LIN and CEF is given in Table 1. The method was found linear (with excellent correlation coefficients) in concentration ranges of 50–2000 ngmL^-1^ for both LIN and CEF (Table 1).

**Table 1:** Validation parameters of HPLC-PDA for the simultaneous determination of LIN and CEF.

Parameters	LIN	CEF
Calibration range (ng mL^− 1^)	50-2000	50-2000
Slope	0.0911	0.0882
Intercept	0.6986	0.2180
Correlation coefficient (r)	0.9998	0.9996
LOD (ng mL^− 1^)	24	21
LOQ (ng mL^− 1^)	43	45

The sensitivity of HPLC-PDA was assessed and expressed as its limits of detection (LOD) and limits of quantitation (LOQ). The LOD and LOQ were calculated according to the ICH Q2 (R2) guideline (2022) based on the signal-to-noise ratio method, where LOD corresponds to a signal-to-noise ratio of 3 and LOQ corresponds to a signal-to-noise ratio of 10 with relative standard deviation values less than 10%^30^. The LOD values were found to be 24 and 21 ngmL^-1^ for LIN and CEF, respectively, while the LOQ values were 43 and 45 ngmL^-1^ for LIN and CEF, respectively. The slight difference in LOQ values reflects inherent differences in molar absorptivity at 230 nm and baseline noise levels at the respective retention times. Specifically, LIN exhibits slightly higher molar absorptivity (ε ~ 1.85 × 10⁴ L·mol⁻¹·cm⁻¹) compared to CEF (ε ~ 1.72 × 10⁴ L·mol⁻¹·cm⁻¹), and the retention time of CEF at 9.2 min experiences slightly higher baseline noise compared to LIN at 4.8 min. Critically, both LOQ values are well below the expected plasma concentrations following therapeutic oral doses (C_max_ ~ 950–1,550 ngmL^-1^), confirming that the method possesses adequate sensitivity for the intended pharmacokinetic application. The LOQ values of both LIN and CEF (43 and 45 ngmL^-1^, respectively) demonstrate adequate sensitivity for quantification in biological fluids, consistent with previously reported bioanalytical methods (LOQ of 50 ngmL^-1^ for LIN in rat plasma^34^ and LOQ of 48 ngmL^-1^ for CEF in human plasma^21^.

#### Selectivity

The specificity assessment involved a comparison of chromatograms derived from three groups of plasma samples. The groups consisted of blank plasma, plasma spiked with LIN, CEF, and PAR, as well as plasma samples from rats administered combined doses of LIN and CEF, subsequently spiked with PAR. The chromatograms (Fig. 1) indicate the absence of peaks from the plasma matrix at the migration times of LIN, CEF, or PAR, thereby confirming the selectivity of the HPLC-PDA method for the simultaneous determination of LIN and CEF in plasma samples.

#### Accuracy and precision

The accuracy and precision of the analytical method were validated by a series of intra-day and inter-day experiments. For both LIN and CEF, five replicate measurements were taken at three distinct quality control concentrations (LQC, MQC, and HQC). The accuracy, calculated as percent bias, exhibited a range of -10.66% to 8.13% for intra-day measurements and − 10.72% to 9.50% for inter-day measurements (Table S3). Precision was quantified by the relative standard deviation (RSD), with intra-day RSDs of 0.220% to 1.229% and inter-day RSDs of 0.289% to 2.881%. The consistently low bias and RSD values demonstrate the high degree of accuracy and precision of the developed HPLC-PDA method. According to ICH M10 (2022) requirements, precision and accuracy were assessed using four quality control concentration levels prepared in rat plasma: LLOQ QC (50 ngmL^-1^, ± 20% of the lower limit of quantitation), Low QC (150 ngmL^-1^, approximately 3× LLOQ), Medium QC (700 ngmL^-1^, approximately 30–50% of the calibration range), and High QC (1500 ngmL^-1^, ≥ 75% of the upper limit of quantitation of 2000 ngmL^-1^). Five replicate measurements were performed for each level in three independent runs. The accuracy, calculated as percent bias, exhibited a range of -10.66% to 8.13% for intra-day measurements and − 10.72% to 9.50% for inter-day measurements (Table S3). Precision was quantified by the relative standard deviation (RSD), with intra-day RSDs of 0.220% to 1.229% and inter-day RSDs of 0.289% to 2.881%. The consistently low bias and RSD values demonstrate the high degree of accuracy and precision of the developed HPLC-PDA method.

#### Stability and extraction recovery

The stability of LIN and CEF was assessed in three concentration levels of plasma samples under various conditions, including bench-top stability at 25 °C for 6 h and three freeze-thaw cycles for 12 h from − 20 °C to room temperature. The recoveries were consistently high, ranging from 97.42 ± 3.901% to 100.12 ± 4.226% for LIN and from 97.85 ± 3.946% to 98.79 ± 2.403% for CEF (Table S4). These findings confirm that the plasma matrix does not affect the stability of either compound in the prepared solutions.

The extraction efficiency was also confirmed, with mean recoveries of 100.20 ± 4.692% for LIN and 99.82 ± 1.519% for CEF (Table S5), validating the effectiveness of the extraction method.

#### Matrix effect

Matrix effect was assessed by comparing the peak areas of LIN and CEF spiked into post-extracted blank plasma from six different lots with those of neat standards at low and high QC concentrations (150 and 1500 ngmL⁻¹). The matrix factor, calculated as the ratio of analyte peak area in plasma to that in neat solution, ranged from 92.3% to 98.7% for LIN and 91.8% to 97.5% for CEF, indicating no significant ion suppression or enhancement. The internal standard-normalized matrix factors were 0.96 ± 0.04 for LIN and 0.95 ± 0.05 for CEF, all within the acceptable range of 0.85–1.15 per ICH M10 guidelines.

#### Carryover

Carryover was evaluated by injecting blank plasma samples immediately after the highest concentration calibration standard (2000 ngmL⁻¹) in triplicate. No detectable peaks exceeding 20% of the LLOQ were observed at the retention times of LIN, CEF, or PAR, confirming that carryover is negligible.

#### Dilution integrity

Dilution integrity was assessed by spiking rat plasma at concentrations above the ULOQ (4000 ngmL⁻¹ for both LIN and CEF) and diluting 5-fold and 10-fold with blank plasma. The accuracy of diluted samples (back-calculated to the original concentration) ranged from 92.5% to 98.3% for LIN and 91.7% to 97.6% for CEF, with RSD values below 5%, confirming that samples above the ULOQ can be reliably diluted while maintaining accuracy and precision.

### Results of pharmacokinetic study

**Fig. 2:**
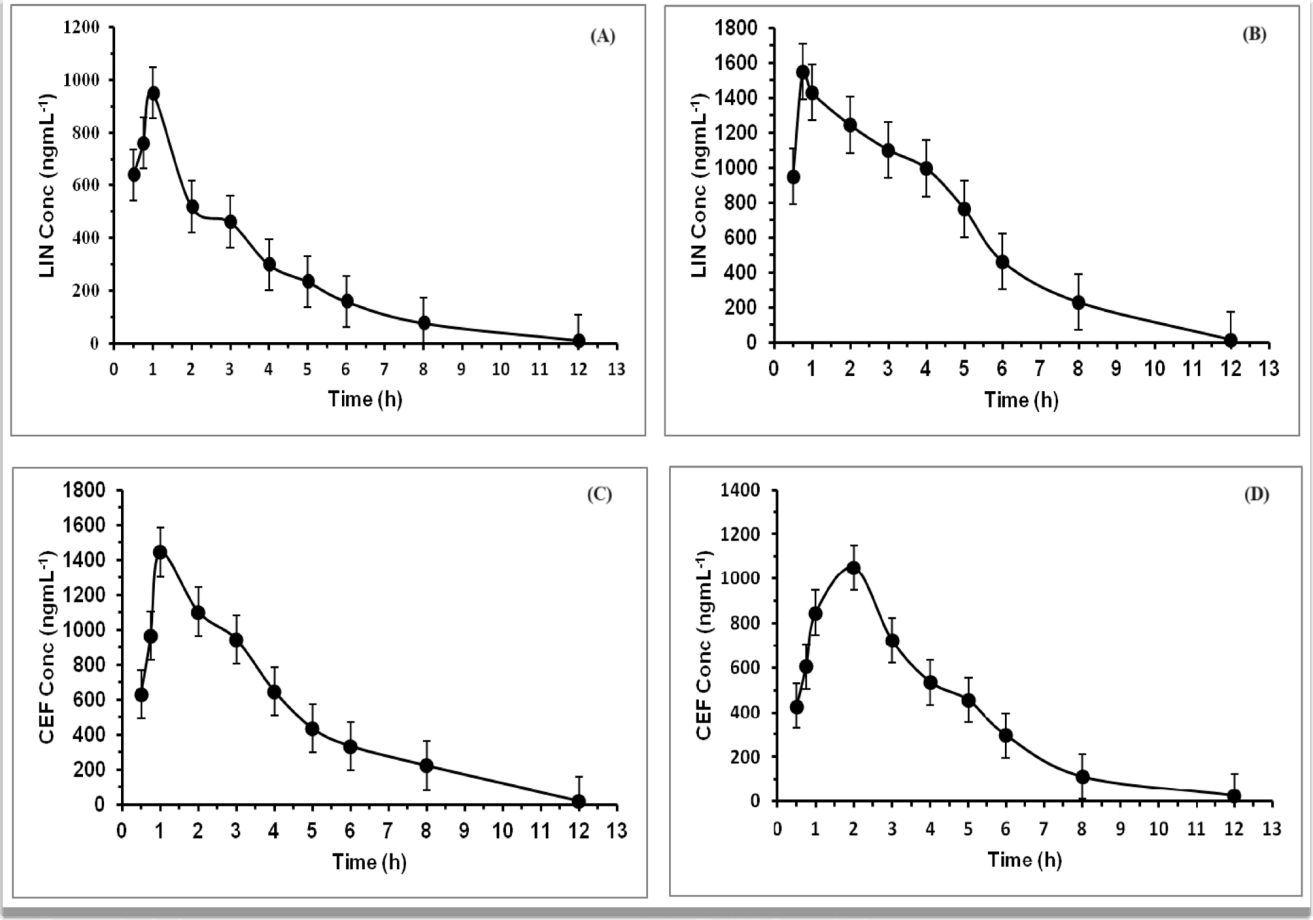
Mean plasma concentration-time profiles of linagliptin and cefixime following oral administration to rats (*n* = 7 per group). (**A**) Linagliptin alone (10 mg/kg). (**B**) Linagliptin (10 mg/kg) co-administered with cefixime (8 mg/kg); AUC increased by 134.2%, C_max_ increased by 63.2%. (**C**) Cefixime alone (8 mg/kg). (**D**) Cefixime (8 mg/kg) co-administered with linagliptin (10 mg/kg); AUC decreased by 23.3%, C_max_ decreased by 27.3%. Data points represent mean ± SD.

In order to assess the combined effects of the medications when administered concurrently, the pharmacokinetic characteristics of the medications that were assessed individually were employed as comparison points. Regression equations were employed to determine the pharmacokinetic characteristics of the administered medications by plotting their plasma concentrations at different time intervals. As a result, a graph was generated to illustrate the average plasma concentration over time, as shown in Fig. 2. PK Solver^45^ was utilized to conduct a non-compartmental pharmacokinetic study (NCA), and the results of all groups were compared as shown in Table 2. A non-compartmental analysis (NCA) approach was selected over compartmental modeling for three primary reasons. First, NCA does not assume a specific compartmental structure, reducing potential bias when assessing drug-drug interactions with unknown mechanistic effects. Second, NCA is the regulatory standard for bioequivalence and drug-drug interaction studies as recommended by both FDA and EMA guidelines. Third, the sampling schedule of ten time points across 12 h provides robust estimates of non-compartmental parameters (AUC, C_max_, t₁/₂) but is insufficient for reliable estimation of compartmental micro-constants, particularly in the presence of potential non-linearities introduced by the drug-drug interaction.

**Table 2:** Results of pharmacokinetic study by using the proposed HPLC-PDA method for determination of LIN and CEF.

parameters	LIN		CEF	
	Group administered with LIN	Group administered with LIN and CEF	Group administered with CEF	Group administered with CEF and LIN
T_max_(h)	1	0.75	1	2
T_1/2_ (h)	1.63	1.24	1.66	1.22
C_max_(ng mL^− 1^)	950.23	1550.54	1445.45	1050.12
Elimination constant (k)	0.43	0.56	0.42	0.57
Vd, (mg)/(ngml^− 1^)) ^a^	0.008	0.002	0.003	0.004
Cl, (mg)/(ngml^− 1^)/h)) ^b^	0.003	0.001	0.015	0.019
AUC 0→t (ngml^− 1^.h)	3038.75	7157.03	5718.37	4403.5
AUC 0→∞ (ngml^− 1^.h)	3066.92	7183.74	5763.83	4421.04

^a^Vd = Volume of distribution.

^b^Cl = Clearance.

By observing the pharmacokinetic curves in Fig. 2, it was noticed that LIN and CEF were rapidly detected in plasma almost after 0.5 h after oral administration in all groups which ease their determination. The time required to reach the maximum plasma absorption for LIN declines by approximately 0.25 h when combined with CEF than when administered alone, whereas for CEF, it was raised by an hour when it was taken simultaneously with LIN in group (Ⅲ).When LIN was combined with CEF, the half-life of LIN was decreased apparently (from 1.63 to 1.24 h); similarly to the half-life of CEF, it was dwindled obviously (from 1.66 to 1.22) which indicated that the drug is being cleared from the body more quickly. In terms of maximum plasma concentration (C_max_), while LIN had increased significantly by 63.15% which indicates higher therapeutic efficacy, CEF had declined enormously by 27.34% which indicates lower therapeutic efficacy. LIN bioavailability was hugely increased by 134.24% increase in LIN plasma levels when administered concurrently with CEF in group (Ⅲ); however, CEF bioavailability was reduced by 23.30% reduction in CEF plasma levels when combined with LIN in group (Ⅲ).The enormous profound change in the bioavailability of the two drugs ascertains that they require dose adjustment or monitoring to maintain therapeutic levels. The volume of distribution (V_d_) of the examined medications were significantly influenced by their contemporaneous administration, with (V_d_) for LIN decreasing by 67.53% which means that it is restricted to the bloodstream with limited ability to penetrate into body tissues in contrast to the (V_d_) of CEF which increased by 33.33%. LIN and CEF clearance (Cl) were influenced as well by their concurrent administration, while (Cl) of LINwas declined by 57.58% which may lead to higher levels of LIN increasing the risk of adverse effects if accumulation occurs, CEF (Cl) was increased by 26.67% which may cause reduced plasma levels and potentially lowering its therapeutic efficacy. This suggests a bidirectional drug-drug interaction where each drug may influence the other’s metabolism or excretion, and dose adjustments or therapeutic monitoring should be needed.

It was obvious that both drugs’ pharmacokinetics were changed substantially, therefore, when they were prescribed concurrently, caution should be considered as there were a massive change in their bioavailabilities. Both drugs require dose monitoring based on the data appeared in their pharmacokinetic parameters which ascertain a severe interaction between LIN and CEF when taken together. Due to a discernible change in their bioavailabilities, this necessitates the regulation of their dose regimen. All pharmacokinetic parameters are given in Table 2.

#### Clinical significance for diabetic patients

The observed bidirectional pharmacokinetic interaction carries direct clinical relevance for diabetic patients receiving LIN and CEF concomitantly. The 63.2% increase in LIN C_max_ and 134.2% increase in LIN AUC, together with a 57.6% reduction in LIN clearance, suggest that diabetic patients particularly those with concomitant renal impairment may face an elevated risk of LIN accumulation and hypoglycemia when CEF is co-administered. Conversely, the 27.3% decrease in CEF C_max_ and 23.3% decrease in CEF AUC, coupled with a 26.7% increase in CEF clearance, raise concerns about suboptimal antibiotic efficacy, especially in severe infections such as diabetic foot infections or complicated urinary tract infections. These findings indicate that dose monitoring or adjustment may be necessary when LIN and CEF are prescribed concurrently in diabetic patients. Confirmatory studies in human subjects are warranted before definitive clinical recommendations can be made.

### Greenness assessment of HPLC-PDA method

Green Analytical Chemistry (GAC) promotes the creation of analytical procedures that are both highly efficient and environmentally benign, particularly for analyzing trace analytes in complex matrices^46^. In line with this philosophy, the greenness of the new HPLC-PDA method was evaluated using four distinct assessment tools: the eco-scale assessment (ESA)^47^, the green analytical procedure index (GAPI)^48^, and analytical greenness (AGREE)^49^ and Blue Applicability Grade Index (BAGI)^50^.

The rationale for employing four distinct assessment tools is that no single metric captures all aspects of greenness comprehensively. The Eco-Scale is quantitative but overlooks practicality. GAPI provides visual communication of environmental impact but remains inherently subjective. AGREE offers a comprehensive, 12-principle evaluation but is relatively complex to calculate. BAGI addresses method applicability and fitness-for-purpose but does not assess environmental impact. Thus, using all four tools together provides a complete, balanced, and complementary assessment of the method’s greenness and practical utility.

#### Eco-scale Assessment (ESA)

The ESA tool evaluates the environmental impact of an analytical procedure by assigning penalty points (PPs) for negative factors, such as solvent use, energy consumption, waste generation and occupational hazards. These points are then subtracted from a perfect score of 100. The proposed HPLC-PDA method achieved a score of 81 PPs (Table 3), which indicates the method’s environmental friendliness.

**Table 3:** Eco-scale penalty points, GAPI and AGREE pictograms of the developed HPLC-PDA method for simultaneous determination of LIN and CEF in spiked rat plasma.

Eco-scale assessment			
Reagents			
Solvents	Amount	Hazard^a^	Total PPs^b^
Methanol	1 (1 < 10 mL)	2 (3pictograms, danger)	6
Sodium phosphate	1 (1 < 10 mL)	1 (0pictograms, danger)	0
Phosphoric acid	1 (1 < 10 mL)	2 (2pictograms, danger)	4
Instruments			
Energy used	1(≤ 1.5 kWh per sample)		
Occupational hazard	0		
Waste	5(~ 12 mL)		
Waste treatment	3(no waste treatment)		
Total PPs	Σ19		
Eco-scale score	81		
**GAPI pictogram**	**AGREE pictogram**	**BAGI pictogram**	
			

^a^Hazard penalty points = No. of pictograms × signal. The signal maybe warning = 1 or danger = 2.

^b^The total penalty points = the amount penalty points ×hazard penalty points.

^c^Total waste volume per injection (~ 12 mL) includes: mobile phase waste (0.8 mLmin⁻¹ × 12 min = 9.6 mL), methanol used for protein precipitation (1 mL per sample), and solvent used for standard preparation (1 mL per sample).

#### Green Analytical Procedure Index (GAPI)

The GAPI tool provides a detailed, 15-parameter assessment of the entire analytical process (Table 3). For the proposed method, the majority of the parameters were colored green or yellow, signifying an environmentally sound procedure. However, a single red color was assigned to parameter 1 due to the requirement for offline sample preparation and storage at -20 °C.

#### Analytical Greenness (AGREE)

The AGREE metric evaluates analytical methodologies based on 12 parameters (Table 3) and presents the results in a colored circular pictogram. The HPLC-PDA method demonstrated strong adherence to the principles of Green Analytical Chemistry (GAC), achieving a final score of 0.64 out of 1.

#### Blue Analytical Method (BAGI)

The newly introduced BAGI metric provides a quantitative assessment of an analytical method’s “blueness,” which relates to its practicality and fitness for purpose^50^. Based on 10 crucial factors, the method was assigned a score of 82.5/100 (Table 3), which indicates its high relevance and functionality for the intended application.

In summary, the combined results from the ESA, GAPI, AGREE, and BAGI assessments confirm that the proposed HPLC-PDA method is both environmentally friendly and highly suitable for the routine analysis of LIN and CEF in plasma samples, successfully meeting the criteria of Green Analytical Chemistry.

## Conclusion

This study describes the development and validation of a new HPLC-PDA method for the simultaneous quantification of LIN and CEF in rat plasma. The method offers sufficient sensitivity, enabling accurate determination of both analytes at low nanogram which was 43 and 45 ngmL^-1^ for LIN and CEF, respectively. Sample pretreatment involved a simple and rapid protein precipitation step using methanol without the need for complex liquid–liquid or solid-phase extraction, facilitating high-throughput analysis suitable for clinical settings. Chromatographic separation of LIN, CEF, and the internal standard (IS) was achieved within a short run time of less than 12 min, with distinct retention times ensuring baseline resolution. The extraction recovery rates ranged from 96.63% to 105.53% and 98.85% to 101.57% for LIN and CEF, respectively (with RSD values of 4.692% and 1.519% for LIN and CEF, respectively). The method demonstrated excellent accuracy (≥ 89.34% and 91.31% for LIN and CEF, respectively) and precision (RSD ≤ 0.289% and 0.501% for LIN and CEF, respectively), supporting its application in streamlining sample detection in pharmacokinetic and bioequivalence studies. Importantly, when LIN and CEF were co-administered, a significant pharmacokinetic interaction was observed: LIN plasma concentrations maximized, while CEF concentrations decreased. This interaction may have clinical relevance and warrants caution during co-prescription, particularly in patients with co-morbid conditions especially diabetics.

Environmental sustainability of the method was evaluated using four established greenness assessment tools: the Analytical Eco-Scale score was 81/100), GAPI score was 6/15, AGREE score was 0.64/1, and BAGI score was 82.5/100. These results confirm that the method adheres to green analytical chemistry principles without compromising analytical performance. The validated method provides a reliable platform for further investigation of LIN-CEF interactions and supports therapeutic drug monitoring in complex clinical scenarios.

Several limitations of the present method should be acknowledged. The method was developed and validated only in rat plasma; extrapolation to human plasma requires additional validation. Potential interference from commonly co-administered medications (e.g., metformin, statins, anti-hypertensives) was not evaluated and should be addressed in future selectivity studies. Additionally, this represents a single-laboratory validation, and the pharmacokinetic findings require confirmation in humans before clinical dose adjustments can be recommended.

## Supplementary Information

Below is the link to the electronic supplementary material.

Supplementary Material 1

## Data Availability

The datasets used and/or analyzed during the current study are available from the corresponding author on reasonable request.
