# Supplementary material for: Green HPLC-PDA method for simultaneous determination of linagliptin and cefixime with pharmacokinetic application in rats
Source: Sci Rep. 2026 Jul 7;16:21011. doi: 10.1038/s41598-026-57925-0 (PMC13342621; doi:10.1038/s41598-026-57925-0)
Supplement: Supplementary file 1 — Supplementary Material 1 [file 41598_2026_57925_MOESM1_ESM.docx]

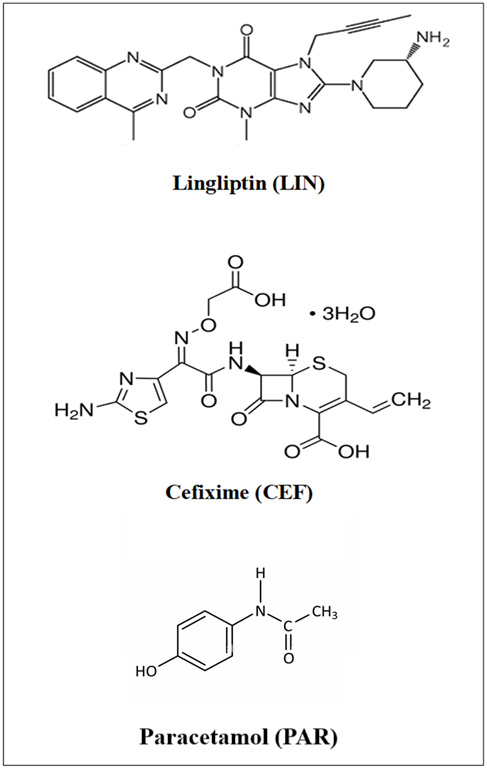


**Fig.S1** The chemical structures of Linagliptin (LIN), Cefixime (CEF) and Paracetamol (PAR).

**
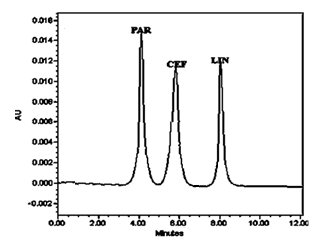
**

**Fig.S2** A representative chromatogram of standard solution containing cefixime, linagliptin and the internal standard paracetamol. The concentrations of cefixime, linagliptin and paracetamol were 800, 750, 1000 ngmL^-1^, respectively. AU is the detector response in arbitrary units.

**Table S1.** Optimization of experimental conditions for the HPLC-PDA for the simultaneous determination of LIN and CEF

| Parameter investigated | Studied range | Optimum value |
| --- | --- | --- |
| Buffer pH (unit) | 3- 6 | 4.3 |
| Buffer concentration (mM) | 10-30 | 20 |
| Scanning wavelength (λ) | 230, 245,254,270,290 | 230 |
| Flow rate (mL/min) | 0.4-1.2 | 0.8 |

**Table S2.** System suitability parameters for the performance characteristics of HPLCE-PDA method for the simultaneous determination of LIN and CEF.

|  | **HPLC-PDAmethod** | | | | | | |
| --- | --- | --- | --- | --- | --- | --- | --- |
|  | **Plasma** | **PAR** | | **CEF** | | **LIN** | **Referencevalue** [39] |
| **Retentiontime(t_R_)(min)** | 1.98 | 3.95 | | 5.64 | | 7.96 | **-** |
| **Resolution(R_s_)** | 4.38 | | 3.76 | | 5.16 | | >1.5 |
| **Selectivity(α)** | 8.95 | | 1.86 | | 1.63 | | >1 |
| **Tailingfactor(T)** | - | 1.00 | | 1.00 | | 1.00 | ≈1 |
| **Capacityfactor(K')** | - | 1.00 | | 1.85 | | 3.02 | >1 |
| **Column efficiency(N)** | - | 998.56^a^ | | 2035.814 | | 4055.142 | Increasewith efficiency  oftheseparation |
| **HETP(cm**/**plate)** | - | 2.50×10^-2^ | | 1.23×10^-2^ | | 6.20×10^-3^ | The smaller the value, the higher the column efficiency |

^a^The lower plate count for paracetamol (998.56) is due to its early elution (3.95 min) and is acceptable because resolution between all critical peak pairs (PAR/CEF = 3.76, CEF/LIN = 5.16) exceeds USP requirements, and the analytes of interest (LIN and CEF) demonstrate excellent efficiency (4,055 and 2,036 plates, respectively).

**Table S3**. The intra– and inter–day precision and accuracy of the proposed HPLC-PDA for the simultaneous determination of LIN and CEF in spiked rat plasma samples.

| Concentration (ng mL^-1^) | Intra–day | | |  | Inter–day | | |
| --- | --- | --- | --- | --- | --- | --- | --- |
|  | Recovery (%) ^a^ | RSD (%) ^a^ | Bias (%) ^b^ |  | Recovery(%) ^a^ | RSD (%) ^a^ | Bias (%) ^b^ |
| LIN |  |  |  |  |  |  |  |
| 50 | 96.32 | 0.531 | -3.68 |  | 94.40 | 1.233 | -5.60 |
| 150 | 97.03 | 1.175 | -2.97 |  | 96.92 | 1.628 | -3.08 |
| 700 | 89.34 | 0.226 | -10.66 |  | 89.28 | 0.291 | -10.72 |
| 1500 | 90.08 | 0.220 | -9.92 |  | 96.13 | 0.289 | -3.87 |
| CEF |  |  |  |  |  |  |  |
| 50 | 98.21 | 0.543 | -1.79 |  | 97.47 | 0.773 | -2.53 |
| 150 | 101.03 | 0.813 | 1.03 |  | 101.24 | 1.064 | 1.24 |
| 700 | 91.31 | 0.851 | -8.69 |  | 99.35 | 0.887 | -0.65 |
| 1500 | 94.14 | 0.463 | -5.86 |  | 94.10 | 0.501 | -5.90 |

^a^ Average of 3 determinations.

^b^ Bias = [(measured concentration - nominal concentration)/nominal concentration] × 100. Values are an average of 3 determinations.

**Table S4.** Stability results of LIN and CEF in spiked rat plasma at different conditions using the proposed HPLC-PDA.

| Concentration  (ng mL⁻¹) | Recovery^a^ | | | |
| --- | --- | --- | --- | --- |
|  | **Bench-top stability (25°C, 6 hours)** | **Three freeze-thaw cycles**  **(-20°C → RT)^b^** | **Long-term stability**  **(-20°C, 30 days)** | **Post-preparative stability (4°C autosampler, 24 hours)** |
| LIN |  |  |  |  |
| 150 | 100.40 | 101.56 | 98.45 | 99.03 |
| 700 | 98.72 | 99.34 | 97.23 | 98.12 |
| 1500 | 93.14 | 103.45 | 96.89 | 97.56 |
| Mean | 97.42 | 100.12 | 97.52 | 98.24 |
| RSD (%) | 3.901 | 4.226 | 0.801 | 0.756 |
| CEF |  |  |  |  |
| 150 | 100.86 | 102.22 | 97.34 | 98.45 |
| 700 | 96.20 | 96.45 | 96.56 | 97.89 |
| 1500 | 99.32 | 94.89 | 95.89 | 96.78 |
| Mean | 98.79 | 97.85 | 96.60 | 97.71 |
| RSD (%) | 2.403 | 3.946 | 0.75 | 0.856 |

^a^Average of 3 determinations. ^b^ RT: Room temperature, Freezing was done at –20 ºC.

**Note:** Whole blood stability was not evaluated as samples were centrifuged immediately after collection (within 15 minutes) per standard pharmacokinetic practice.

**Table S5**. The extraction recovery of LIN and CEF from spiked rat plasma.

| **Concentration (ng mL^-1^)** | **Recovery (%) ^a^** |  |
| --- | --- | --- |
|  | **LIN** | **CEF** |
| **150** | 105.53 | 101.57 |
| **700** | 96.63 | 98.85 |
| **1500** | 98.45 | 99.05 |
| **Mean** | 100.20 ± 4.692 | 99.82 ± 1.519 |

^a^ Average of 3 determinations.
